# Terminology missing distinction and implication: Nuances of physiological and performance durability

**DOI:** 10.1007/s00421-026-06294-7

**Published:** 2026-06-02

**Authors:** Lars Schwalm, Marcelle Schaffarczyk, Dominik Fohrmann, Karsten Hollander, Thomas Gronwald

**Affiliations:** 1 Institute of Interdisciplinary Exercise Science and Sports Medicine, MSH Medical School Hamburg, Am Kaiserkai 1, 20457 Hamburg, Germany; 2 Integrative and Experimental Exercise Science and Training, Institute of Sport Science, Julius-Maximilians-Universität Würzburg, Würzburg, Germany

With regard to the aim of critically examining and conceptually clarifying terminology surrounding the emerging construct of durability, we read with great interest the Viewpoint and the subsequent Last Word on the Viewpoint entitled “Durability, fatigability, repeatability, and resilience in endurance sports: definitions, distinctions, and implications” by Meixner et al. (2025a, b). We believe that the current construct of durability provides sufficient conceptual coverage, whereas additional constructs risk introducing redundancy and ambiguity. Nevertheless, we appreciate the authors’ efforts to emphasize the importance of achieving semantic and conceptual coherence within this evolving field (Meixner et al. 2025b).

We perceive that the ongoing discussion has largely focused on physiological mechanisms and methodological considerations related to the assessment rather than terminology (Faude et al. 2025; Black et al. 2025). The focus of this comment is stepping back to more fundamentally address the semantic clarity and conceptual necessity of the proposed constructs. The concepts of *durability*,* fatigability*,* repeatability*, and *resilience* are inherently interconnected (Meixner et al. 2025a), reflecting related physiological and psychological capacities that contribute to performance in a context-dependent manner across sport-specific, individual, and environmental demands along the laboratory-field continuum.

We therefore consider it necessary to comment on this ongoing discussion. The current construct of *durability* already provides conceptual coverage and the proposed definitions do not sufficiently acknowledge existing conceptual overlap. Consequently, these overlapping definitions introduce ambiguity and increase the risk of misinterpretation, particularly in applied settings, while also hindering mechanistic interpretation and comparability across studies. This commentary specifically addresses semantic clarity and construct necessity, rather than mechanisms or measurement specificities.

Durability: The term *durability* has been defined as the “deterioration of physiological characteristics over time during prolonged exercise” (Maunder et al. 2021; Schwalm et al. 2025), a definition also acknowledged by Meixner et al. (2025a). Hunter et al. (2025) distinguish between *physiological and performance durability*. In this framework, the authors included prolonged endurance events lasting more than 60 min, specifically in human endurance performance contexts (Hunter et al. 2025). *Physiological durability* refers to physiological demands and is characterized by the magnitude and timing of the decoupling between internal (e.g., heart rate, oxygen consumption) and external load (pace, power) during prolonged exercise. In contrast, *performance durability* pertains to external load metrics and is operationalized by maximal mean power or pace, or the corresponding power– or pace–duration profile, assessed after a defined amount of work has been completed. Nevertheless, it should be considered that this framework is limited to a specific subset of endurance events (i.e., those > 60 min) and may not be generalizable.

Fatigability: Meixner et al. (2025a) characterize *fatigability* as the “(in)ability to repeat efforts at workloads corresponding to or near mean maximal power output without rest”. They explicitly acknowledge that exercise intensity during competition is inherently variable (Meixner et al. 2025a). In this context, Maunder et al. (2021) note that the capacity to repeatedly generate periods of higher demand and to recover during periods of lower demand within a task, race, or competition is dynamic rather than static. However, the distinction from prolonged endurance exercise remains unclear. In long-distance running, repeated maximal efforts alone are rarely decisive for successful performance. Instead, prolonged events typically involve continuous pace maintenance, progressive pace increases, intermittent surges, and end spurts, often influenced by drafting dynamics or undulating terrain. Under such stochastic exercise conditions, repeated high intensity demands and the progressive decoupling of internal and external load occur simultaneously, making a clear conceptual separation between *fatigability* and *durability* difficult. Consequently, repeated surges in exercise intensity during stochastic pacing may induce the same cumulative physiological strain that *durability* aims to describe.

Repeatability: *Repeatability* was defined as the “capacity to recover and reproduce high-intensity performance across multiple bouts, stages, or heats with passive recovery in between” (Meixner et al. 2025a). A conceptual distinction from *durability* may be justified when exercise tests or race efforts are repeated under systematically different conditions. However, the underlying principle remains closely related to *performance durability*, which was previously also defined as the change in performance between fresh and fatigued states (Hunter et al. 2025). Repeated bouts separated by recovery periods still involve accumulated physiological strain and subsequent performance reproduction, suggesting substantial conceptual overlap between *repeatability* and *performance durability*. In applied settings such as multiple competitions within a single day or repeated sessions within a micro-cycle, *repeatability* may therefore be better viewed as a context-specific manifestation or extension of *performance durability* rather than as a fully distinct construct.

Resilience: The inclusion of the term *resilience*, defined as the “capacity to maintain or regain physiological and mechanical function under conditions of fatigue, environmental extremes, or other perturbations” (Meixner et al. 2025a), warrants further conceptual clarification. In psychological research, *resilience* is typically understood as a dynamic process or trait reflecting the ability to adapt to or recover from adversity (Troy et al. 2023). However, there is an ongoing debate regarding its precise definition and operationalization. This perspective differs from a physiological interpretation, where *resilience* may be framed as the stability or recovery capacity of biological subsystems under stress (Jones 2023).

While such a physiological framing is plausible, its application in the context of exercise physiology appears to overlap substantially with the concept of *durability*. Particularly, when referring to the ability to resist fatigue and maintain performance over time. Importantly, there are influencing factors operating at the intra-individual level (e.g., pre-condition, hydration status, substrate availability) and environmental conditions (e.g., heat, hypoxia). These may be more appropriately considered as contextual modifiers of performance and the decoupling of external-to-internal load rather than defining features of a distinct construct (Gronwald et al. 2020). As outlined previously (Hunter et al. 2025), these factors should be considered independently of whether exercise demands are prolonged or intermittent, and across the entire laboratory-field continuum. Taken together, *resilience* overlaps conceptually in performance contexts with *durability* and does not represent a distinct construct.

In addition to conceptual considerations, we would like to emphasize that semantic clarity and simplicity are necessary in science and have applied implications for communication between researchers and practitioners. Therefore, the ongoing debate highlights a broader issue: the increasing divergence between scientific terminology and applied practice, where multiple constructs with partially overlapping meanings are used to describe similar phenomena. The introduction of additional terms may create challenges for clear communication between coaches, athletes, and scientists. For example, a progressive increase in heart rate or oxygen consumption at a constant power output during prolonged exercise represents a clear indicator of reduced physiological durability (i.e., internal-to-external load decoupling), yet may be misinterpreted in practice as impaired “*fatigability*”. In contrast, a measurable decline in maximal mean power or pace following a defined amount of prior work reflects reduced *performance durability*, but may be incorrectly attributed to a lack of “*repeatability*” or described more generally as poor “*durability*” without specification. Conversely, an inability to reproduce high-intensity efforts across repeated bouts with recovery is more appropriately classified as repeatability, rather than a limitation in durability per se.

These one-to-one mismatches between observation and terminology illustrate how insufficient differentiation between *physiological durability*,* performance durability*, and other proposed constructs can lead to incorrect interpretation of physiological and performance indicators and, consequently, to inappropriate training emphasis. Within the scope of this comment, these examples are intended to demonstrate the practical relevance of maintaining precise conceptual boundaries between constructs. In particular, using terms with opposing value directions—where higher *durability* is viewed as advantageous and higher *fatigability* is seen as undesirable—may contribute to confusion rather than improve conceptual clarity. This directional meaning (good vs. bad) creates ambiguity in communication in applied settings.

Overall, we agree with Meixner et al. (2025a) that a critical discussion of key concepts and terminology is both timely and necessary. These considerations highlight the relevance of raising awareness regarding the inconsistent use and incomplete definition of emerging constructs proposed as extensions to the classical three-dimensional endurance model (Joyner and Coyle 2008; Jones 2023). The current discussion suggests that constructs such as *fatigability*,* repeatability*, and *resilience* (Meixner et al. 2025a) still require further conceptual and operational refinement before they can be considered as clearly distinct constructs. Until clearer boundaries are established, *durability* may currently be best viewed as the overarching construct, encompassing context-dependent expressions such as *repeatability*. From this perspective, *physiological and performance durability* may provide the most primary constructs moving forward. At the same time, the emergence of overlapping constructs is common in evolving subfields, and we appreciate the effort to enhance semantic coherence before inconsistencies become more deeply embedded in applied and scientific practice.

As endurance research continues to shift from rigid, laboratory-based approaches toward more context-specific, real-world environments, the construct of *durability* indeed helps capture performance variability that the traditional models cannot fully explain. However, as proposed by Meixner et al. (2025b), meaningful progress requires linguistic and conceptual alignment before mechanistic exploration can be pursued. Without shared prototypes and agreed-upon definitions, measurement will remain arbitrary, and cumulative scientific progress will be limited. To bridge this gap between science and practice, we support initiating an international expert consensus process to establish shared terminology and clearly defined conceptual boundaries for this emerging construct. A practical way forward could involve a Delphi process with invited experts from different regions and fields of application (Beato et al. 2026), similar to previous position statements in exercise physiology and sports medicine.
